# Glycosylation Patterns in *Meccus (Triatoma) pallidipennis* Gut: Implications for the Development of Vector Control Strategies

**DOI:** 10.3390/microorganisms13010058

**Published:** 2025-01-01

**Authors:** Elia Torres-Gutiérrez, Frida Noelly Candelas-Otero, Olivia Alicia Reynoso-Ducoing, Berenice González-Rete, Mauro Omar Vences-Blanco, Margarita Cabrera-Bravo, Martha Irene Bucio-Torres, Paz María Silvia Salazar-Schettino

**Affiliations:** Departamento de Microbiología y Parasitología, Facultad de Medicina, Universidad Nacional Autónoma de México, México City 04510, Mexico

**Keywords:** triatomine gut, glycosylation patterns, vector control, Chagas disease vector

## Abstract

The primary mode of transmission for Chagas disease is vector-borne transmission, spread by hematophagous insects of the *Triatominae* subfamily. In Mexico, the triatomine *Meccus pallidipennis* is particularly significant in the transmission of *Trypanosoma cruzi*. This study focused on analyzing protein expression and modifications by glycosylation in different regions of the digestive tract of fifth-instar nymphs of *M. pallidipennis*. Two gut sections were dissected and extracted: the anterior midgut (AMG) and the proctodeum or rectum (RE). Proteins were extracted from each tissue sample and profiled by one- and two-dimensional electrophoresis; protein glycosylation was analyzed by lectin affinity. Our results showed significant differences in protein expression and glycosylation between both gut regions, with modifications being more frequent in the RE. The proteins HSP70, actin, and tubulin were analyzed, finding a differential expression of the latter two between AMG and RE. Understanding glycosylation patterns provides critical insights into vector–pathogen interactions that could eventually inform novel control approaches. Furthermore, the potential use of lectins as insecticidal agents highlights the broader implications of glycoprotein research in the future development of strategies on vector control to disrupt *T. cruzi* transmission.

## 1. Introduction

Triatomines (*Triatominae*) are a subfamily of hematophagous hemipterans that are considered potential vectors of *Trypanosoma cruzi*, the causative agent of Chagas disease. Transmission to vertebrates occurs when the insect deposits feces containing the parasite during or after feeding, which allows its entry to the body by being carried to the bite site, through wounds or mucous membranes, or even through the skin. This is the natural mechanism of infection and the most common in humans in countries such as Mexico, where it is considered a neglected disease [1]. Chagas disease is a chronic, systemic disorder characterized by cardiac involvement, with cardiomegaly and/or megaly in digestive organs, with potential for disability or death. It is endemic in 21 countries in the Americas, where it is associated with poor socioeconomic conditions [2]. In Mexico, an estimated 4 million people are infected [3].

In Mexico, *Meccus pallidipennis* is a major vector of *T. cruzi* in animal and human reservoirs [4]; all nymphal and adult stages of *M. pallidipennis* and other triatomine species are strictly hematophagous, and they can be infected by and transmit *T. cruzi*. Feeding is critical for their development, as body wall distension can trigger molting to the next stage [5,6]. *M. pallidipennis* is mainly distributed in the states of Colima, Jalisco, Michoacán, Morelos, and Guerrero, where it is the main vector of the disease [5,7]. In Sanitary Jurisdiction 2 of Morelos (Jojutla), it is reported as a peridomiciliary pest [8]; however, in some regions of Guerrero [9] and Morelos, the vector has been described as intradomiciliary, with natural infection rates up to 79.7% [10]. This vector, along with six other species in the Phyllosoma complex, is responsible for approximately 74% of vector transmission of *T. cruzi* in Mexico [11].

The digestive system of triatomines is responsible for blood digestion and nutrient absorption. The tract is divided into the anterior midgut (AMG), posterior midgut (PMG), and proctodeum or rectum (RE) [12]. Several types of proteins are expressed in the gut, including heat shock proteins and proteases, which are important for cell protection and hemoglobin digestion [13,14]. Proteins involved in oxidative stress response, innate immunity, and cellular metabolism, which are important for the insect’s adaptation to its environment and interaction with *T. cruzi*, are also expressed [14,15].

In the AMG, blood is stored, coagulation is inhibited, and erythrocytes are lysed by enzymatic processes; this is where most of the bacterial microbiota is located, which multiplies after blood ingestion. *T. cruzi* enters the triatomine in the blood of an infected vertebrate and suffers high mortality in this region [16,17]. Most of the digestive and nutrient absorption processes take place in the PMG, and it is here that trypomastigotes differentiate into epimastigotes and reproduce [16]. Finally, water and mineral salts are absorbed in the RE, and it is here that epimastigotes differentiate into metacyclic trypomastigotes found in the insect’s feces and urine, which are the infective form for vertebrates [12]; for this reason, the RE is a key target for controlling disease transmission.

Glycosylation, a fundamental process in these insects, adds carbohydrate chains to proteins and lipids to form glycoconjugates (glycoproteins and glycolipids) [18]. This occurs mainly in the endoplasmic reticulum and the Golgi apparatus. In the former, newly synthesized proteins bind to glycan structures that are enzymatically modified on their way to the Golgi apparatus [19].

Although most glycosylation studies in insects have been conducted in *Drosophila melanogaster*, it has been shown that glycans, composed of monosaccharides such as fucose, galactose, and N-acetylglucosamine, play critical roles in metabolism, cell signaling, and immune response, and are involved in a variety of biological processes ranging from development to nervous system function and insect longevity. Therefore, interfering with glycosylation in insects could be a promising strategy for their control [18,20,21,22]. Recent studies have identified insect-specific glycans, suggesting special evolutionary adaptations that could be useful biomarkers in control strategies by disrupting glycosylation processes and affecting vector development and survival [22]. In insects, up to 90% of O-glycans are known to be of the mucin type, containing N-acetylgalactosamine (GalNAc) attached directly to serine or threonine residues; they are abundant in secreted proteins or in the extracellular domains of membrane proteins and are involved in important processes such as recognition, adhesion, signaling, and immunity [22]. The study of glycosylation patterns is relevant to the transmission of *T. cruzi* in nature. The influence of carbohydrates on parasite adhesion to the insect PMG was demonstrated by inhibiting parasite adhesion after incubation with N-acetylgalactosamine, N-acetylmannosamine, N-acetylglucosamine, D-galactose, D-mannose, or sialic acid [23]. Other researchers have described differences in *M. pallidipennis* gut protein profiles depending on diet and *T. cruzi* infection [24,25]. Regarding the detected modifications by glycosylation, they were mainly increased in fed insects [26]. However, despite the known differences in the function of triatomine gut regions, their glycosylation patterns have not been investigated. In this work, the glycosylation patterns of the AMG and RE of *M. pallidipennis* fifth-instar nymphs were determined with the aim of finding differences and identifying sensitive targets for the development of biological control strategies of triatomines, thus contributing to the interruption of vectorial transmission of Chagas disease, a priority goal of the WHO.

## 2. Materials and Methods

### 2.1. Obtaining Insects

Thirty freshly molted, unfed, fifth-instar nymphs of *M. pallidipennis* were randomly selected from the insectary of the Laboratory of Parasite Biology, Department of Microbiology and Parasitology, Faculty of Medicine, UNAM. The insects were maintained at 28 °C, 60% humidity, and 12 h light/dark cycles.

### 2.2. Tissue Dissection and Extraction

Insects were dissected in Petri dishes at 4 °C under a stereomicroscope; limbs were removed with dissecting forceps and the abdomen was disinfected with swabs soaked in 70% alcohol. The connexivum of each insect was removed to expose the gut, and the anterior midgut (AMG) and rectum (RE) were identified and removed. The posterior midgut (PMG) was excluded from the study as it is underdeveloped in fasting insects. The organs were placed separately in 1.5 mL Eppendorf tubes, labeled, and washed with 1 mL sterile PBS, pH 7.2. Phosphate buffer with protease inhibitors (cOmplete, Roche, Basel, Switzerland) was added to each tube until all tissues were covered. Tissue samples were stored at −80 °C until processing

### 2.3. Protein Extraction

Tissue samples from 30 fifth-instar nymphs were slowly thawed and 100 μL of phosphate buffer containing protease inhibitors (cOmplete, Roche, Basel, Switzerland) was added. Cell disruption was performed in three round of sonication (Branson Ultrasonics™ Sonifier™ SFX150 Cell Disruptor). For the AMG, this process was performed in three intervals at 15% amplitude for 15 s, alternating with 60 s cooling on ice/ethanol. For the RE, two 20 s intervals were applied, the first at 20% amplitude and the second at 30% amplitude. This procedure was adapted from the methodology described by Ambrosio et al. (2003) [27] and further reported in studies [28,29].

Protein extracts pooled from 30 different insects were precipitated in 10% trichloroacetic acid [30] and 20 mM 1,4-bis(sulfanyl)butane-2,3-diol (dithiothreitol, DTT) in cold acetone and solubilized in phosphate buffer pH 7.4 with protease inhibitors. Proteins were quantified by the bicinchoninic acid (BCA) method [31] using a Pierce BCA kit (Thermo Scientific™, Waltham, MA, USA, Cat. No. 23225) according to the supplier’s directions.

### 2.4. Protein Profiling by SDS-PAGE Electrophoresis (1D)

Protein profiling of the different regions of *M. pallidipennis* gut was performed by electrophoresis (SDS-PAGE) [32] on premade 4–12% gradient polyacrylamide gels (NuPAGE™ 4–12% Bis-Tris Gel, 1.0 mm × 12 well, Invitrogen, Waltham, MA, USA, Ref. NP0322BOX, Thermo Fisher Scientific, Waltham, MA, USA) with 20 μg protein per well and broad molecular weight markers (10–250 kDa) (Precision Plus Protein™ Dual Color Standards, Cat. 1610374, Bio-Rad, Hercules, CA, USA) in an electrophoresis chamber (Mini Gel Tank, Invitrogen™ Thermo Fisher Scientific) at 200 V for 45 min. The gel was stained with Coomassie Blue (R-350). 

### 2.5. Proteomic Mapping by Isoelectric Focusing and Two-Dimensional (2D) Electrophoresis

For isoelectric focusing, 7 cm immobilized gradient (IPG) strips with a linear 3–10 pH range (IPG Strips, Bio-Rad, Hercules, CA, USA; Cat. 1632000) were used. Briefly, 80 μg of protein from each gut region was loaded onto each strip with rehydration solution (7M urea, 2M thiourea, 4% CHAPS, 60 mM DTT, 2% IPG buffer [Cytiva; Cat. 17600087], and 0.002% bromophenol blue). The samples, which required urea, were maintained at room temperature (around 20 °C) and never exceeded 37 °C to avoid carbamylation. The run was performed on a PROTEAN i12 IEF cell kit (Bio-Rad) in four steps, (1) 250 V/15 min, (2) 4000 V/60 min, and (3) 4000 V, (4) until 15 000 V/h was reached. Once focused and equilibrated, the strips were loaded onto a premade 4–12% gradient polyacrylamide gel (NuPAGE™ 4–12% Bis-Tris ZOOM™ Gel, 1.0 mm × IPG well, Invitrogen; Cat. NP0330BOX) and run in an electrophoresis chamber at 200 V for about 45 min. Gels were stained with colloidal Coomassie [33].

### 2.6. Glycosylation Patterns from Lectin Blots

After 1D or 2D electrophoresis, the gels were transferred to polyvinylidene fluoride (PVDF) membranes (Immobilon^®^-P, Merck Millipore, Burlington, MA, USA; Cat. IPVH00010) in a transfer chamber (Transfer-Blot Turbo™, Bio-Rad) at 15 V/30 min. For glycosylation detection, the membranes were first blocked in 0.3% PBS-Tween 20-0.1% albumin with the lectins ConA (peroxidase-conjugated concanavalin A from *Canavalia ensiformis*) (Sigma-Aldrich, St. Louis, MO, USA, Cat. L6397) at a dilution of 0.5 μg/μL, WGA (peroxidase-conjugated wheat germ agglutinin from *Triticum vulgaris*) (Sigma-Aldrich, Cat. L3892) at a dilution of 0.5 μg/μL, and PNA (peroxidase-conjugated peanut agglutinin from *Arachis hypogaea*) (Sigma-Aldrich, Cat. L7759) at a dilution of 1 μg/μL. This was followed by seven washes with PBS-0.3% Tween 20-0.1% Triton x100, seven washes with PBS-0.3% Tween 20, and seven washes with PBS pH 7.2. The reaction was visualized with 2 mM 3,3-diaminobenzidine (DAB) and 0.01% hydrogen peroxide (H_2_O_2_) [34].

### 2.7. Protein Detection by Western Blot

After electrophoretic separation of 20 μg protein per well and transfer, the membranes were incubated for 60 min at room temperature in blocking solution with the following primary antibodies at 1:1000 dilution: DM1A (anti-α-tubulin mouse monoclonal, Sigma Life Science, Cat. No. T6199-200UL), RM112 (anti-β-actin rabbit monoclonal Sigma Aldrich, Cat. No. SAB5600204), and HSP70 (anti-heat shock protein mouse monoclonal Sigma Aldrich, Cat. No: SAB4200714-100UL). The membranes were washed three times with PBS-0.3% Tween 20 for 5 min. Secondary antibodies for DM1A and HSP70 were mouse anti-IgG at 1:1000 dilution (goat pAb to Ms IgG/HRP, abcam, Cat. No. ab97040); for RM112 it was rabbit anti-IgG at 1:2000 dilution. Reactions were developed with 2 mM DAB (Bio-Rad) and 0.01% H_2_O_2_ in PBS pH 7.2 [35].

### 2.8. Gel and Transfer Analysis

The 1D and 2D gels and PVDF membranes were imaged using a gel documentation system (Gel Doc™ XR Imaging System, Bio-Rad Molecular Imager^®^). Molecular weight determination of proteins in 1D gels and transfers and band analysis were performed using the software ImageLab 6.1, Bio-Rad. The resulting masters were analyzed by a comparative proteomics tool using PD Quest 7.0.4 software (Bio-Rad), which allowed us to assign false colors (green and red) to the protein spots from each sample, overlaying the maps to produce a single map with green, red, and yellow spots. Yellow spots represent proteins present in both samples, facilitating the identification of shared and unique proteins.

### 2.9. Statistical Analysis

The molecular weight and isoelectric point were obtained for the points detected in the proteomic maps. Proteins were classified as unique or shared between gut regions. The data were plotted and analyzed by Student’s *t*-test.

From glycosylation patterns, the frequency of components identified by molecular weight and isoelectric point for each lectin was plotted against its respective proteomic map.

In addition, the points on each map were grouped and quantified by gut sector and displayed graphically in a heat map.

The relative reaction intensity of Western blot assays was determined semiquantitatively by volume analysis using the software Image Lab 6.1 (Bio-Rad).

## 3. Results

### 3.1. Protein Quantification in the Gut of Meccus Pallidipennis N5 Nymphs

**Figure 1:**
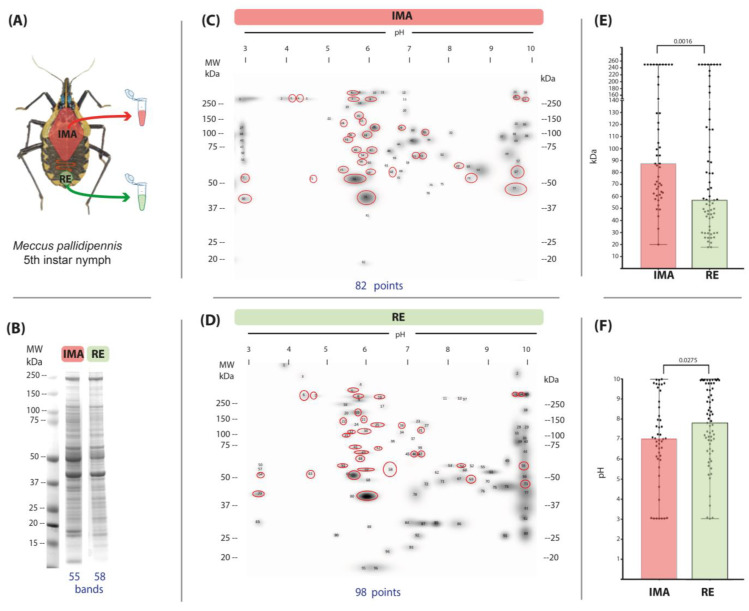
Protein profiles and protein maps of the anterior midgut (AMG) and rectum (RE) of unfed, fifth-instar nymphs of *M. pallidipennis*. (**A**) Schematic of regions excised from *M. pallidipennis* gut. (**B**) Protein profiles of the AMG and RE on SDS-PAGE; MW = molecular weight, kDa = kilodaltons. Total bands identified in each gut section are shown at the bottom. (**C**) Master proteomic map of the AMG. (**D**) Master proteomic map of the RE. Red circles indicate common points in both maps. The total number of detected points is shown at the bottom of each map. (**E**) Distribution of unique points from each gut region by molecular weight (kDa); *p* < 0.0001. (**F**). Distribution of unique points from each gut region by isoelectric point; *p* < 0.0001.

The two gut regions dissected from the insects differ significantly in size (Figure 1A), which is reflected in the amount of protein obtained from each region. Over 80% of the total protein extracted from this samples was derived from the AMG, while only 18% corresponded to the RE (Table 1).

### 3.2. Proteomic Profiling and Mapping of the Anterior Midgut (AMG) and Rectum (RE) of Meccus Pallidipennis N5 Nymphs

Both gut regions showed bands with a wide range of molecular weights, from >250 kDa to <15 kDa; the expression profile was similar in the two gut regions analyzed. Fifty-five bands were detected in the AMG and fifty-eight in the RE (Figure 1B). The bands of highest intensity in both regions had molecular weights of 241, 56, and 42 kDa; in addition, bands of 18, 17, and 11 kDa were prominent in the AMG (Figure 1B).

Different protein expression patterns were found in the proteomic maps of the AMG and RE of *M. pallidipennis* N5 nymphs, with 34 conserved spots in both regions. Eighty-two spots were detected in the AMG, of which forty-eight were unique (Figure 1C). In the RE, 98 spots were detected, of which 64 were unique (Figure 1D). When the distribution of unique spots in each region was analyzed by molecular weight (MW, kDa) (Figure 1E) and isoelectric point (IP) (Figure 1F), statistically significant differences were observed. In the RE, as opposed to the AMG, there was a greater expression of unique spots between 50 and 15 kDa and with IPs between 5 and 10, in addition to the presence of spots with MW > 250 kDa, which were less abundant in the AMG (Figure 1E,F).

### 3.3. Glycosylation with α-Mannose and α-Glucose in the AMG and RE

**Figure 2:**
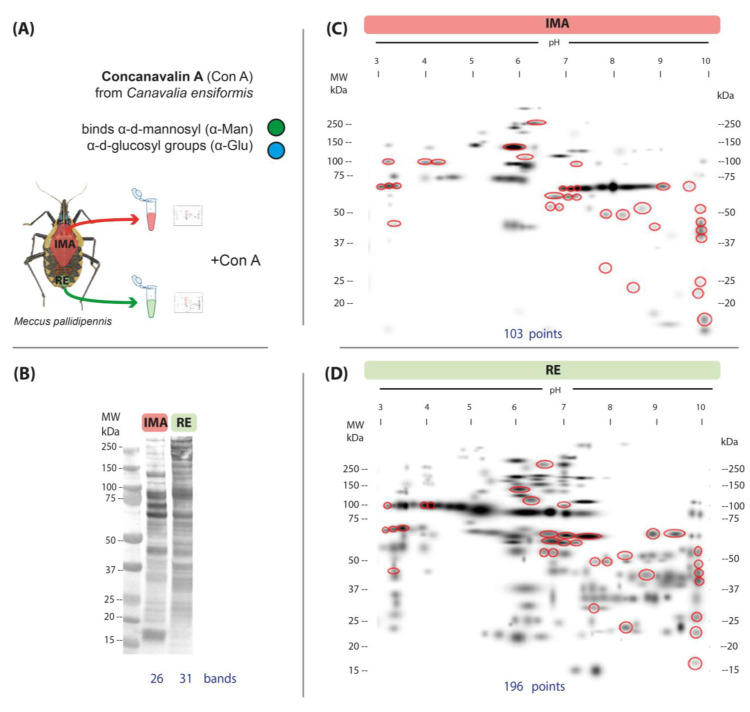
Glycosylation patterns of α-mannose and α-glucose detected with concanavalin A (ConA) in the anterior midgut (AMG) and rectum (RE) of unfed, fifth-instar nymphs of *M. pallidipennis*. (**A**) Schematic of regions excised from *M. pallidipennis* gut for glycan detection with ConA. (**B**) Glycosylation profiles of the AMG and RE by 1D lectin blot. The total number of glycosylation bands identified in each gut section is shown at the bottom. (**C**) Master map of affinity to ConA in the AMG. (**D**) Master map of affinity to ConA in the RE. MW = molecular weight, kDa = kilodaltons. Red circles indicate common glycosylated spots in both samples. The total number of spots identified in each gut region is shown at the bottom of each map.

Glycosylated bands were detected in 1D lectin blots of the AMG and RE (Figure 2A). Modifications with mannose and glucose were detected by affinity to Con A; 26 bands were recognized in the AMG and 31 in the RE. Three visible labeled bands with molecular weights of 87, 67, and 45 kDa were found in both gut regions (Figure 2B).

In 2D lectin blots, 34 common spots with α-mannose and/or α-glucose glycosylation were detected in both gut regions. In the AMG, 103 spots were detected, of which 69 were unique (Figure 2C); in the RE, 196 spots were observed, of which 162 were unique (Figure 2D).

### 3.4. Glycosylation with N-Acetylglucosamine and Sialic Acid in the AMG and RE

**Figure 3:**
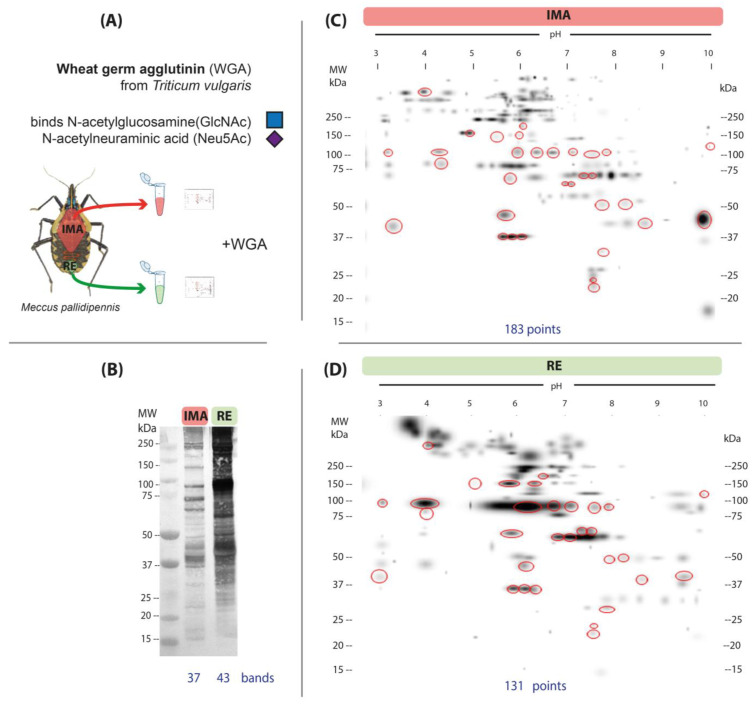
N-acetylglucosamine and sialic acid glycosylation patterns detected by WGA in the anterior midgut (AMG) and rectum (RE) of unfed, fifth-instar nymphs of *M. pallidipennis*. (**A**) Schematic of regions extracted from *M. pallidipennis* gut for glycan detection with WGA. (**B**) Glycosylation profiles of AMG and RE by 1D lectin blot. The total number of glycosylation bands identified in each section of the intestine is shown at the bottom. (**C**) Master map of affinity to WGA in the AMG. (**D**) Master map of affinity to WGA in the RE. MW = molecular weight, kDa = kilodaltons. Red circles indicate common glycosylated spots in both samples. The total number of spots identified in each gut region is shown at the bottom of each map.

Glycosylation with N-acetylglucosamine and sialic acid was detected by affinity with the WGA lectin in the AMG and RE (Figure 3A). In 1D lectin blots, 37 bands were detected in the AMG and 43 in the RE (Figure 3B). On the other hand, the AMG showed a higher number of glycosylation spots (183 spots) in 2D maps (Figure 3C) compared to the RE, where 131 spots were identified (Figure 3D). There were 150 and 98 unique and 33 common spots identified in the AMG and RE, respectively.

### 3.5. Glycosylation Maps with N-Acetylgalactosamine and β-Galactose in the AMG and RE

**Figure 4:**
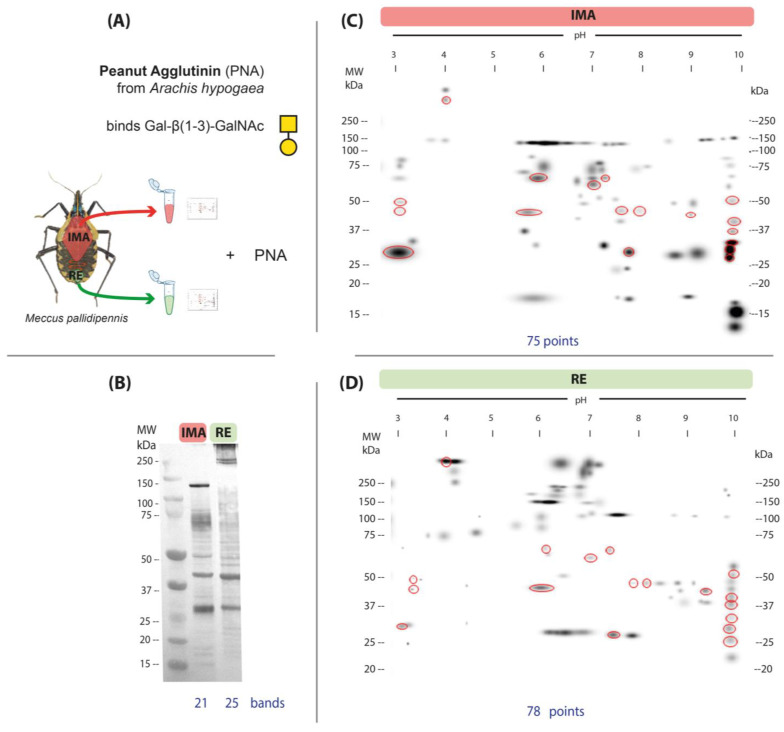
Glycosylation patterns of N-acetylgalactosamine and β-galactose by peanut agglutinin (PNA) in the anterior midgut (AMG) and rectum (RE) of unfed, fifth-instar nymphs of *M. pallidipennis*. (**A**) Schematic of regions extracted from *M. pallidipennis* gut for glycan detection with PNA. (**B**) Glycosylation profiles of the AMG and RE by 1D lectin blot. The total number of glycosylation bands identified in each section of the gut is shown at the bottom. (**C**) Master map of PNA affinity in the AMG. (**D**) Master map of affinity for PNA in the RE. MW = molecular weight, kDa = kilodaltons. Red circles indicate common glycosylated spots in both samples. The total number of spots identified in each gut region is shown at the bottom of each map.

The presence of N-acetylgalactosamine and galactose in the AMG and RE was detected by PNA (Figure 4A). In the AMG, 21 bands were observed, among which the 134 kDA band is notable for its intensity and definition. In the RE, 25 bands were identified (Figure 4B). The two regions share 15 bands, with the 41.8 kDa band being the most intense.

Protein glycosylation maps obtained by lectin blot with PNA showed 75 spots in the AMG (Figure 4C) and 78 spots in the RE (Figure 4D); however, the patterns in both gut regions were different, with only 14 common spots, 57 unique spots in the AMG, and 60 unique spots in the RE. The AMG showed a higher number of unique proteins with MW < 37 kDa, whereas several spots with MW > 250 kDa were observed in the RE (Figure 4C,D).

### 3.6. Distribution of Protein and Glycosylation Spots in the AMG and RE

**Figure 5:**
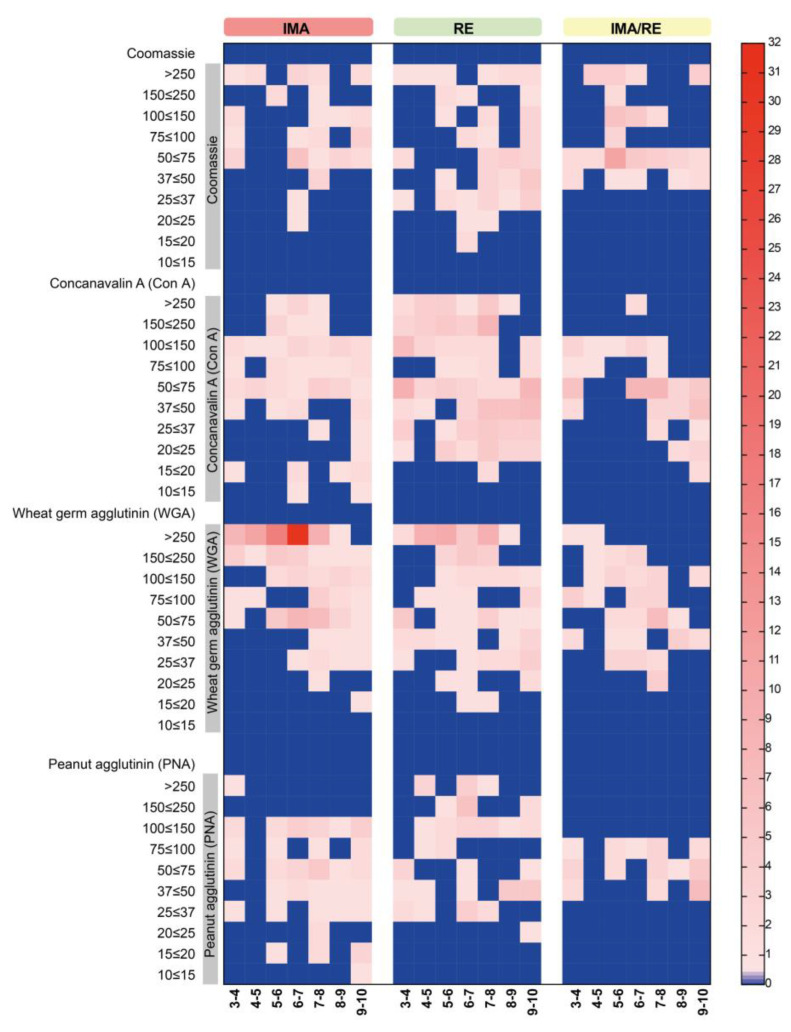
Differential expression patterns of protein and glycosylation spots in the AMG and RE, and spots in common (AMG/RE). Protein spots identified in the 2D electrophoresis master maps and glycosylation spots identified in the affinity master maps for the lectins ConA, WGA, and PNA were grouped and quantified by defined isoelectric point (X-axis) and molecular weight (Y-axis) sectors and plotted on a heat map. Red and blue shades represent higher and lower levels of protein expression and glycosylation, respectively.

Both protein and glycosylation spots were found in the AMG and RE of unfed N5 nymphs of *M. pallidipennis* with the three lectins used. The distribution of these spots by molecular weight and isoelectric point showed differences between the two gut regions. The differential patterns of protein expression and glycosylation are shown in a heat map (Figure 5).

According to the observed patterns, the common protein spots between AMG and RE have MW > 37 kDa and isoelectric points ranging from 3 to 10.

Spots indicating glycosylation with mannose (ConA) were more abundant and more widely distributed in the RE than in AMG (Figure 2C,D). Glycosylation with N-acetylglucosamine/sialic acid (WGA) was more frequent in the AMG than in the RE (Figure 3C,D); however, the reactions were more intense in the RE. In addition, it was noteworthy that glycosylation detected with this lectin occurred in numerous high-molecular-weight proteins (>250 kDa) in both the AMG and RE. Glycosylation with N-acetylgalactosamine (PNA) was less frequent in both the AMG and RE and showed marked differences in distribution patterns, with lower-molecular-weight spots in the AMG and MW > 250 kDa in the RE (Figure 4C,D).

### 3.7. Differentially Expressed Proteins in the AMG and RE

**Figure 6:**
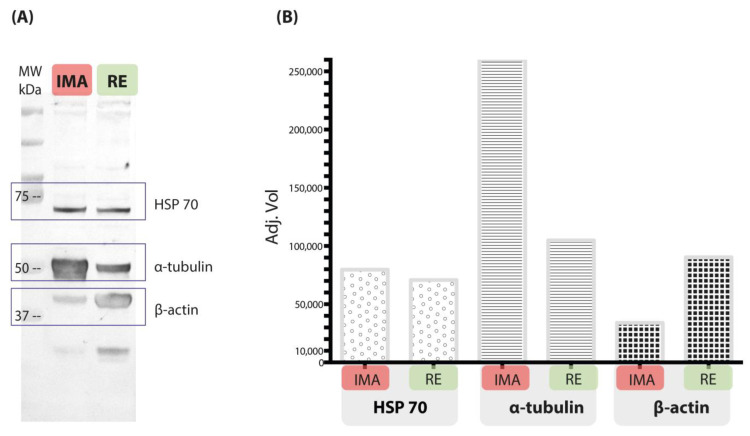
Relative expression of proteins detected in the anterior midgut (AMG) and rectum (RE) of unfed, fifth-instar nymphs of *M. pallidipennis*. (**A**) Immunodetection of the proteins HSP70 (BRM-22), α-tubulin (DM1A), and β-actin (RM112) by Western blot. MW = molecular weight, kDa = kilodalton. (**B**) Histogram of the semiquantitative evaluation of reaction intensity detected by Western blot (Image Lab, Bio-Rad).

Expression of the heat shock protein HSP70 was detected in both the AMG and RE by Western blot. The band corresponding to this protein was well defined in both gut regions (Figure 6).

The structural proteins α-tubulin and β-actin were identified in both gut regions. However, the band intensity of α-tubulin was higher in the AMG than in the RE, while β-actin showed a higher expression in the RE (Figure 6).

## 4. Discussion

The gut of triatomine insects plays a crucial role in the transmission of Chagas disease, being both the primary site of blood digestion and the site of interaction with the parasite. The development of *T. cruzi* in the digestive tract of insects is an essential step in its life cycle [15]. In this study, proteomic profiles, glycosylation maps, and protein characteristics of different regions of the gut of *M. pallidipennis* were determined.

The proteomic patterns in the anterior midgut (AMG) and rectum (RE) showed little variation, with 55 and 58 bands, respectively, higher than the 12 bands previously reported in a whole gut analysis [26]. However, the proteomic maps revealed significant differences in the distribution of molecular weight (MW) and isoelectric point (PI) between the 82 protein spots identified in the AMG and the 98 in the RE. These differences indicate distinct protein expression profiles in both regions, aligning with findings from a recent review that identified 82 proteins expressed exclusively in the AMG, 17 in the PMG, and 2 in the RE [36]. While direct comparisons of 2D gels without normalization standards are challenging, the use of specialized software provided a reliable approach to emphasize the differences and similarities between these two regions.

Studies on proteomic maps of triatomine intestines are scarce, mainly focusing on the AMG [13,24]. When comparing the AMG proteomic map obtained in this work with that of *Rhodnius prolixus*, a significant difference was observed in the number of spots identified, with 475 analyzed spots in Vieira’s work [13]. Despite this quantitative difference, at least 12 spots showed similarities in MW and IP, suggesting that they may correspond to structural proteins essential for the function of the AMG.

On the other hand, 295 protein spots were reported in the AMG of fed *M. pallidipennis* females [24], with similarities in the pattern of some spots with this study, suggesting the presence of constitutive proteins. In addition, Nava’s map showed more protein spots with MW < 37 kDa, which could be related to proteins involved in food processing and digestion [24]. Similarly, other researchers described remarkable differences in the AMG proteomic maps of fed *M. pallidipennis* males in terms of the number and distribution of protein spots [25].

It is evident that the differences in the proteomic maps reported in this study in unfed N5 nymphs and previous reports in *R. prolixus* and *M. pallidipennis* may be due to factors such as the developmental stage of the triatomines, feeding conditions, extraction methods, and the amount of protein quantified in each study.

Protein glycosylation is a highly complex process that occurs mainly in the endoplasmic reticulum and the Golgi apparatus and involves a variety of enzymes (glycosyltransferases and glycosidases) as well as activated sugars, whose availability and concentration are closely linked to the cellular energy status [37]. Therefore, environmental factors such as fasting or feeding may influence protein glycosylation patterns. In this study, we worked with unfed, fifth-instar (N5) nymphs of *M. pallidipennis*. Despite limited nutrient availability, abundant glycosylated protein bands and spots were observed, with significant differences in glycosylation patterns between gut regions.

In this study, glycosylation was detected by lectins due to their high affinity and potential use in insect control, as demonstrated in previous studies [20,21]. Carbohydrates found in the gut of triatomines play a crucial role in the development of *T. cruzi* in infected insects. As highlighted in the introduction, the study by Alves et al. (2007), conducted on fifth-instar nymphs, demonstrated that carbohydrates influence parasite adhesion to the insect PMG, with adhesion being inhibited after incubation with N-acetylgalactosamine, N-acetylmannosamine, N-acetylglucosamine, D-galactose, D-mannose, or sialic acid [23]. This underscored the importance of analyzing glycosylation patterns to better understand the transmission of *T. cruzi* in nature.

In this study, a comparison of 1D and 2D lectin blots revealed that some bands observed in the 1D blots did not have corresponding spots in the 2D patterns. This discrepancy may be partially attributed to the distinct sample preparation protocols required for each technique.

The reaction with the lectin ConA, which has an affinity for oligomannose-type N-glycans, was abundantly detected in the RE. N-glycosylation occurs in both the Golgi apparatus and the endoplasmic reticulum and is very common in plasma membrane and secretory proteins involved in biological processes such as adhesion, cell communication, and transmembrane transport [38]. However, previous studies have reported that this type of glycosylation is also common in intracellular proteins involved in vital processes, such as proteases, hydrolases, and phosphatases [18]. Therefore, it is likely that the proteins detected by this lectin in the gut of *M. pallidipennis* are associated with a variety of biological functions.

The identification of mannose-rich glycosylated proteins in the gut of *M. pallidipennis* is particularly relevant because lectins with affinity for mannose such as that of *Galanthus nivalis* (common snowdrop) have been reported to exhibit insecticidal activity by binding to key proteins in metabolic processes, including ferritin and an intestinal aminopeptidase [39,40]. Therefore, it is critical that future studies use mass spectrometry to identify specific glycoproteins and continue the search for potential targets for the biological control of triatomines.

On the other hand, the lectin WGA, which binds to N-acetylglucosamine (GlcNAc) and N-acetylneuraminic acid/terminal sialic acid residues in N- and O-glycans [41], detected glycoproteins predominantly in the AMG. Sialic acid present in mucosa is found at lower levels in the RE, possibly due to the activity of sialidase enzymes that prevent blood coagulation by removing these residues. Although the low levels of sialic acid in the RE do not affect the development of *T. cruzi*, they do explain the low levels of this carbohydrate in parasites isolated from insects [42].

The lectin PNA, which binds to galactose β1,3 N-acetylgalactosamine (Gal β1,3 GalNAc) found in extracellular matrix glycoproteins, was also used to identify glycoproteins [43]. These glycoproteins may be involved in the tissue organization of salivary glands or intestines. This glycosylation type was less abundant, and no differences were observed in the number of spots detected between the AMG and RE, although significant differences were found in their distribution by MW and IP. Previous studies have shown that lectins such as *Rhizoctonia solani* agglutinin (RSA), which binds to GalNAc/Gal, have entomotoxic activity acting on midgut cells [20], suggesting the need to investigate their potential value in triatomine control.

When comparing the different gut regions, the RE showed a higher number of protein bands glycosylated with the three lectins. The glycosylated proteins in this region are probably involved in excretion and contraction processes. Since the RE is fundamental in the life cycle of *T. cruzi*, being the site where metacyclogenesis occurs, it is possible that the glycoproteins detected in this region are involved in the interaction between the parasite and the vector. However, further studies in infected insects are needed to confirm this hypothesis.

The proteins and glycosylation modifications identified using various lectins in the gut of unfed, fifth-instar nymphs may play a role in signaling processes triggered by food deprivation and in preparation for molting into adult males or females. However, further investigations under different feeding conditions and at various developmental stages are necessary to identify the specific proteins and glycosylation types involved in these processes. This study provides a critical foundation for such future research, offering valuable insights to guide subsequent analyses.

Building on the methodology established in this work, and incorporating improvements informed by the lessons learned, future studies will aim to deepen our understanding of these mechanisms. Protocols are already in development to compare unfed, fed, and infected insects, as well as to explore potential differences between sexes in adult insects, where sex differentiation is feasible. These planned investigations represent the next steps in expanding our knowledge of these biological processes.

Initially, selecting specific protein spots of interest for identification was planned to deepen this analysis. Unfortunately, this was not feasible due to the limited proteomic and genetic data available for triatomine species other than *Rhodnius prolixus* [36]. This lack of comprehensive database currently restricts the identification of proteins in species such as *M. pallidipennis* using standard proteomic approaches. We acknowledge the significance of this step and remain hopeful that future expansions in triatomine genetic and proteomic databases will enable the pursuit of this line of investigation.

Structural proteins such as actin and tubulin are constitutively expressed in eukaryotic organisms and play essential roles in cellular maintenance. These proteins are commonly used as endogenous controls to normalize gene expression studies [44]. In this study, the presence of some of these constitutive proteins was assessed using specific antibodies. The α, β, and γ isoforms of actin are the most abundant proteins in eukaryotic cells and are involved in fundamental functions such as cell shape determination, cytokinesis, cytoplasmic streaming, phagocytosis, intracellular signaling muscle contraction, and cell and chromosome movement [44,45]. Another key cytoskeletal protein that plays a fundamental role in maintaining cell structure and intracellular motility is tubulin. In this work, differences in the expression levels of these two proteins were detected, with higher expression of α-tubulin in the AMG and β-actin in the RE. This is in contrast to what was reported by Paim (2012), which validated the use of genes for these proteins as reference controls for qRT-PCR [46]; however, it should be noted that the presence of mRNA does not necessarily indicate that these proteins are expressed.

It has been documented that β-actin is present in most cells as an integral component of the cytoskeleton. In the presence of myosin motors, β-actin networks exhibit numerous foci of contraction [47]. Therefore, the increased presence of β-actin in the RE could be associated with the process of urine and feces excretion.

On the other hand, heat shock proteins (HSPs) are known to be rapidly synthesized in cells in response to environmental stressors [48]. The nymphs of *M. pallidipennis* used in this study were subjected to prolonged fasting, a stress-inducing factor, and showed uniform expression of heat shock proteins in the AMG and RE. The HSP70 family of proteins is highly conserved and probably the best characterized in insects, and it plays a critical role in protecting cellular functions. These proteins act as chaperones, ensuring the proper folding of nascent proteins, minimizing protein aggregation, and facilitating the removal of denatured proteins [15,49].

Actins, tubulins, and HSPs play essential roles in living organisms, so disrupting their synthesis and expression has a serious impact on the development and mortality of organisms, as demonstrated in the *Aedes aegypti* mosquito by reducing the expression levels of tubulin and HSP83 by RNA interference [50].

The detection by Western blot of these two structural proteins and the stress protein suggests the existence of differential protein expression between the AMG and RE in fifth-instar nymphs of *M. pallidipennis*. These results raise new questions as to which other proteins might show significant differences in their expression in different gut regions and which of them might be potential targets for the development of control strategies using specific inhibitors, such as antibodies or RNA interference, or generalized ones, such as lectins.

## 5. Conclusions

This study highlights the complexity of protein expression and glycosylation patterns in different regions of the digestive tract *of Meccus pallidipennis*, with direct implications for vector biology and the life cycle of *Trypanosoma cruzi*. Proteomic profiling of the anterior midgut (AMG) and rectum (RE) revealed significant differences in protein distribution patterns with respect to molecular weight and isoelectric point, suggesting functional specialization in both gut regions. These differences in protein expression highlight the importance of continuing these investigations, considering factors such as developmental stage and feeding conditions in the study of triatomines.

The identification of glycosylation patterns using lectins suggests the presence of glycoproteins critical to biological processes such as cell adhesion and signaling, potentially playing a key role in the interaction between the vector and *T. cruzi*. Glycans on midgut glycoproteins may serve as attachment sites for the parasite, influencing its ability to colonize and develop in specific gut regions. For instance, the abundant mannose-rich glycosylations observed in the RE, associated with excretion and contraction processes, might contribute to the metacyclogenesis of the parasite.

While the specific identities of the detected glycoproteins remain unknown, their differential glycosylation patterns suggest distinct biological roles in the midgut. This study provides a foundation for future proteomic analyses to characterize these glycoproteins and their specific glycosylation patterns. Understanding these profiles offers valuable insights into the molecular interactions between *T. cruzi* and its insect vector, paving the way for identifying molecular targets to disrupt parasite–vector interactions.

Potential applications of these findings include targeting glycoproteins with lectins, glycosidase inhibitors, or other glycan-binding agents to impair *T. cruzi* colonization. Lectins with affinity for mannose, such as those from *Galanthus nivalis*, or for GalNAc/Gal, such as *Rhizoctonia solani* agglutinin (RSA), could serve as promising tools to interfere with key metabolic processes in triatomines. Additionally, lectins themselves have demonstrated insecticidal properties in other studies, presenting a potential dual-action strategy against both the vector and the parasite.

Finally, the differential detection of structural proteins, such as β-actin and tubulin, provides new chances to explore other proteins with essential functions in triatomines. These findings offer opportunities to develop innovative control methods, including the specific inhibition of proteins.

## Figures and Tables

**Table 1:** Protein yield, concentration, and proportion by region from lysates of the anterior midgut (AMG) and rectum (RE) obtained from 30 fifth-instar nymphs of *Meccus pallidipennis*.

Gut Region	Wet Weight (μg)	Protein Concentration (μg/μL)	Total Protein (μg)	Protein by Fifth-Instar Nymph (μg)	Protein Yield (%)	Proportion of Protein by Region (%)
**AMG**	200,000	3.91	5283.90	176.13	2.64	81.30
**RE**	530,000	4.31	1215.70	40.52	0.23	18.70
	730,000	--	6499.60	216.65	0.89	100.00

## Data Availability

All data generated in this study are available within this article.

## References

[1] WHO Neglected Tropical Diseases—Global. https://www.who.int/health-topics/neglected-tropical-diseases.

[2] WHO Chagas Disease (American Trypanosomiasis). https://www.who.int/health-topics/chagas-disease.

[3] Arnal A., Waleckx E., Rico-Chávez O., Herrera C., Dumonteil E. (2019). Estimating the Current Burden of Chagas Disease in Mexico: A Systematic Review and Meta-Analysis of Epidemiological Surveys from 2006 to 2017. PLoS Negl. Trop. Dis..

[4] Salazar Schettino P.M., de Haro Arteaga I., Cabrera Bravo M. (2005). Tres Especies de Triatominos y Su Importancia Como Vectores de Trypanosoma Cruzi En México. Medicina.

[5] Alcantara S.A. (2016). Prevalencia de Trypanosoma Cruzi en Triatoma Pallidipennis en tres Jurisdicciones Sanitarias del sur del Estado de México.

[6] Noireau F., Dujardin J.-P. (2010). Biology of Triatominae. American Trypanosomiasis Chagas Disease (First Edition) One Hundred Years of Research.

[7] Salaza-Schettino P.M., Rojas-Wastavino G.E., Cabrera-Bravo M., Bucio-Torres M.I., Martínez-Ibarra J.A., Monroy-Escobar M.C., Rodas-Retana A., Guevara-Gómez Y., Vences-Blanco M.O., Ruiz Hernández A.L. (2010). Revisión de 13 especies de la familia Triatominae (Hemiptera: Reduviidae) vectores de la enfermedad de Chagas, en México. J. Selva Andina Res. Soc..

[8] Bautista N.L., De La Torre G.S.G., Arteaga I.D.H., Salazar Schettino P.M. (1999). Importance of *Triatoma Pallidipennis* (Hemiptera: Reduviidae) as a Vector of *Trypanosoma Cruzi (Kinetoplastida: Trypanosomatidae)* in the State of Morelos, Mexico, and Possible Ecotopes. J. Med. Entomol..

[9] Rodríguez-Bataz E., Nogueda-Torres B., Rosario-Cruz R., Martínez-Ibarra J.A., Rosas-Acevedo J.L. (2011). Triatomines (Hemiptera: Reduviidae) vectors of Trypanosoma cruzi Chagas 1909, in the State of Guerrero, Mexico. Rev. Biomed..

[10] García-Mares J.I., González-Acosta C., Peralta-Rodríguez J., Correa-Morales F., Barón-Olivares H., Moreno-García M. (2022). Incremento de Incidencia Intradomiciliar de Triatominos y Prevalencia de Trypanosoma Cruzi En El Centro de México. AZM.

[11] Martínez-Ibarra J.A., Nogueda-Torres B., García-Benavídez G., Vargas-Llamas V., Bustos-Saldaña R., Montañez-Valdez O.D. (2012). Bionomics of Populations of *Meccus Pallidipennis* (Stål), 1872 (Hemiptera: Reduviidae) from Mexico. J. Vector Ecol..

[12] Kollien A., Schaub G. (2000). The Development of Trypanosoma Cruzi in Triatominae. Parasitol. Today.

[13] Vieira L.R., Polomé A., Mesquita R.D., Salmon D., Braz G.R.C., Bousbata S. (2015). Protein 2DE Reference Map of the Anterior Midgut of the Blood-Sucking Bug *Rhodnius Prolixus*. Proteom..

[14] Ouali R., Vieira L.R., Salmon D., Bousbata S. (2021). Early Post-Prandial Regulation of Protein Expression in the Midgut of Chagas Disease Vector Rhodnius Prolixus Highlights New Potential Targets for Vector Control Strategy. Microorganisms.

[15] Ouali R., Valentim de Brito K.C., Salmon D., Bousbata S. (2020). High-Throughput Identification of the Rhodnius Prolixus Midgut Proteome Unravels a Sophisticated Hematophagic Machinery. Proteomes.

[16] Schaub G.A., Meiser C.K., Balczun C., Mehlhorn H. (2011). Interactions of Trypanosoma Cruzi and Triatomines. Progress in Parasitology.

[17] Ferreira R.C., Kessler R.L., Lorenzo M.G., Paim R.M.M., Ferreira L.D.L., Probst C.M., Alves-Silva J., Guarneri A.A. (2016). Colonization of *Rhodnius Prolixus* Gut by *Trypanosoma Cruzi* Involves an Extensive Parasite Killing. Parasitology.

[18] Vandenborre G., Smagghe G., Ghesquière B., Menschaert G., Nagender Rao R., Gevaert K., Van Damme E.J.M. (2011). Diversity in Protein Glycosylation among Insect Species. PLoS ONE.

[19] Varki A., Kornfeld S. (2022). Historical Background and Overview. Chapter 1. Essentials of Glycobiology [Internet].

[20] Hamshou M., Van Damme E.J.M., Caccia S., Cappelle K., Vandenborre G., Ghesquière B., Gevaert K., Smagghe G. (2013). High Entomotoxicity and Mechanism of the Fungal GalNAc/Gal-Specific Rhizoctonia Solani Lectin in Pest Insects. J. Insect Physiol..

[21] Macedo M., Oliveira C., Oliveira C. (2015). Insecticidal Activity of Plant Lectins and Potential Application in Crop Protection. Molecules.

[22] Walski T., De Schutter K., Van Damme E.J.M., Smagghe G. (2017). Diversity and Functions of Protein Glycosylation in Insects. Insect Biochem. Mol. Biol..

[23] Alves C.R., Albuquerque-Cunha J.M., Mello C.B., Garcia E.S., Nogueira N.F., Bourguingnon S.C., de Souza W., Azambuja P., Gonzalez M.S. (2007). Trypanosoma Cruzi: Attachment to Perimicrovillar Membrane Glycoproteins of Rhodnius Prolixus. Exp. Parasitol..

[24] Nava-Mirafuentes I. (2015). Proteoma del Estómago de Meccus Pallidipennis (Reduviidae, Triatominae) Asociado a la Infección por Trypanosoma Cruzi. Master’s Thesis.

[25] Sotelo-Cano J.I., Millán-Vega A., Castañeda-Saucedo E., Flores-Robles D., Zumaquero-Ríos J.L., Martínez-Ibarra J.A., Sierra-Martínez P. (2017). Expresión diferencial de proteínas en el estómago de la chinche Meccus pallidipennis, vector de la enfermedad de Chagas. Tlamati.

[26] Gutiérrez-Cabrera A.E., Zandberg W.F., Zenteno E., Rodríguez M.H., Espinoza B., Lowenberger C. (2019). Glycosylation on Proteins of the Intestine and Perimicrovillar Membrane of *Triatoma* (*Meccus*) *Pallidipennis*, under Different Feeding Conditions. Insect Sci..

[27] Ambrosio J.R., Reynoso-Ducoing O., Hernández-Sanchez H., Correa-Piña D., González-Malerva L., Cruz-Rivera M., Flisser A. (2003). Actin Expression in *Taenia Solium* Cysticerci (Cestoda): Tisular Distribution and Detection of Isoforms. Cell Biol. Int..

[28] Cevallos A.M., Segura-Kato Y.X., Merchant-Larios H., Manning-Cela R., Alberto Hernández-Osorio L., Márquez-Dueñas C., Ambrosio J.R., Reynoso-Ducoing O., Hernández R. (2011). Trypanosoma Cruzi: Multiple Actin Isovariants Are Observed along Different Developmental Stages. Exp. Parasitol..

[29] Reynoso-Ducoing O., Valverde-Islas L., Paredes-Salomon C., Pérez-Reyes A., Landa A., Robert L., Mendoza G., Ambrosio J.R. (2014). Analysis of the Expression of Cytoskeletal Proteins of Taenia Crassiceps ORF Strain Cysticerci (Cestoda). Parasitol. Res..

[30] Deatherage Kaiser B.L., Wunschel D.S., Sydor M.A., Warner M.G., Wahl K.L., Hutchison J.R. (2015). Improved Proteomic Analysis Following Trichloroacetic Acid Extraction of Bacillus Anthracis Spore Proteins. J. Microbiol. Methods.

[31] Smith P.K., Krohn R.I., Hermanson G.T., Mallia A.K., Gartner F.H., Provenzano M.D., Fujimoto E.K., Goeke N.M., Olson B.J., Klenk D.C. (1985). Measurement of Protein Using Bicinchoninic Acid. Anal. Biochem..

[32] Laemmli U.K. (1970). Cleavage of Structural Proteins during the Assembly of the Head of Bacteriophage T4. Nature.

[33] O’Farrell P. (1975). High Resolution Two-Dimensional Electrophoresis of Proteins. J. Biol. Chem..

[34] Gravel P. (2002). Identification of Glycoproteins on Nitrocellulose Membranes Using Lectin Blotting. The Protein Protocols Handbook.

[35] Towbin H., Staehelin T., Gordon J. (1979). Electrophoretic Transfer of Proteins from Polyacrylamide Gels to Nitrocellulose Sheets: Procedure and Some Applications. Proc. Natl. Acad. Sci. USA.

[36] Reynoso-Ducoing O.A., González-Rete B., Díaz E., Candelas-Otero F.N., López-Aviña J.A., Cabrera-Bravo M., Bucio-Torres M.I., Torres-Gutiérrez E., Salazar-Schettino P.M. (2023). Expression of Proteins, Glycoproteins, and Transcripts in the Guts of Fasting, Fed, and Trypanosoma Cruzi-Infected Triatomines: A Systematic Review. Pathogens.

[37] Freeze H., Boyce M., Zachara N. (2022). Glycosylation Precursors Chapter 5. Essentials of Glycobiology [Internet].

[38] Stanley P., Moremen K., Lewis N. (2022). N-Glycans Chapter 9. Essentials of Glycobiology [Internet].

[39] Fitches E., Wiles D., Douglas A.E., Hinchliffe G., Audsley N., Gatehouse J.A. (2008). The Insecticidal Activity of Recombinant Garlic Lectins towards Aphids. Insect Biochem. Mol. Biol..

[40] Sadeghi A., Smagghe G., Proost P., Van Damme E.J.M. (2008). Ferritin Acts as a Target Site for the Snowdrop Lectin (GNA) in the Midgut of the Cotton Leafworm *Spodoptera Littoralis*. Insect Sci..

[41] Abo H., Kume M., Pecori F., Miura T., Matsumoto N., Nishihara S., Yamamoto K. (2022). Disaccharide-Tag for Highly Sensitive Identification of O-GlcNAc-Modified Proteins in Mammalian Cells. PLoS ONE.

[42] Amino R., Serrano A.A., Morita O.M., Pereira-Chioccola V.L., Schenkman S. (1995). A Sialidase Activity in the Midgut of the Insect *Triatoma Infestans* Is Responsible for the Low Levels of Sialic Acid in *Trypanosoma Cruzi* Growing in the Insect Vector. Glycobiology.

[43] Cummings R., Etzler M., Hahn M. (2022). Glycan-Recognizing Probes as Tools. Chapter 48. Essentials of Glycobiology [Internet].

[44] Ruan W., Lai M. (2007). Actin, a Reliable Marker of Internal Control?. Clin. Chim. Acta.

[45] Sodja A., Fujioka H., Lemos F.J.A., Donnelly-Doman M., Jacobs-Lorena M. (2007). Induction of Actin Gene Expression in the Mosquito Midgut by Blood Ingestion Correlates with Striking Changes of Cell Shape. J. Insect Physiol..

[46] Paim R.M., Pereira M.H., Di Ponzio R., Rodrigues J.O., Guarneri A.A., Gontijo N.F., Araújo R.N. (2012). Validation of Reference Genes for Expression Analysis in the Salivary Gland and the Intestine of Rhodnius Prolixus (Hemiptera, Reduviidae) under Different Experimental Conditions by Quantitative Real-Time PCR. BMC Res. Notes.

[47] Heissler S.M., Chinthalapudi K. (2024). Structural and Functional Mechanisms of Actin Isoforms. FEBS J..

[48] Mahroof R., Zhu K.Y., Subramanyam B. (2005). Changes in Expression of Heat Shock Proteins in *Tribolium castaneum* (Coleoptera: Tenebrionidae) in Relation to Developmental Stage, Exposure Time, and Temperature. Ann. Entomol. Soc. Am..

[49] Paim R.M.M., Araujo R.N., Leis M., Sant’anna M.R.V., Gontijo N.F., Lazzari C.R., Pereira M.H. (2016). Functional Evaluation of Heat Shock Proteins 70 (HSP70/HSC70) on Rhodnius Prolixus (Hemiptera, Reduviidae) Physiological Responses Associated with Feeding and Starvation. Insect Biochem. Mol. Biol..

[50] Singh A.D., Wong S., Ryan C.P., Whyard S. (2013). Oral Delivery of Double-Stranded RNA in Larvae of the Yellow Fever Mosquito, *Aedes Aegypti:* Implications for Pest Mosquito Control. J. Insect Sci..

